# Lin28B facilitates the progression and metastasis of pancreatic ductal adenocarcinoma

**DOI:** 10.18632/oncotarget.19578

**Published:** 2017-07-26

**Authors:** Yunchao Wang, Jian Li, Shixiang Guo, Yongsheng Ouyang, Liangyu Yin, Songsong Liu, Zhiping Zhao, Jiali Yang, Wenjie Huang, Huan Qin, Xin Zhao, Bing Ni, Huaizhi Wang

**Affiliations:** ^1^ Institute of Hepatopancreatobiliary Surgery, Southwest Hospital, Third Military Medical University, Chongqing 400038, PR China; ^2^ Department of Pathophysiology and High Altitude Pathology, Third Military Medical University, Chongqing 400038, PR China; ^3^ Department of Hepatobiliary Surgery, Zhujiang Hospital, Southern Medical University, Guangzhou 510000, PR China; ^4^ Department of General Surgery, The First Affiliated Hospital, Soochow University, Jiangsu 215006, PR China

**Keywords:** lin28B, pancreatic ductal adenocarcinoma, prognosis, therapeutic target, EMT

## Abstract

Lin28B, a Lin28 homologue, represses the biogenesis of let-7 microRNAs (miRNAs), has a role in tumorigenesis, and is considered a potential therapeutic target for various human malignancies. However, the associations between Lin28B and the clinical features and outcomes of patients with pancreatic ductal adenocarcinoma (PDAC) remain unclear. In this study, we explored the clinical significance of Lin28B in PDAC and its association with metastasis by examining tissues from patients with PDAC and elucidated the molecular mechanisms using PDAC cell lines. In patients, high Lin28B expression was significantly correlated with high levels of lymphatic metastasis, distant metastasis and a poor prognosis. Furthermore, the multivariate analysis identified Lin28B expression as an independent prognostic factor in patients. In cell lines, stable silencing of Lin28B inhibited cell proliferation, cell cycle transition, migration and the epithelial-mesenchymal transition (EMT). It also increased the expression of the c-MYC, HMGA2 and KRAS genes, which are targeted by the cancer-suppressor miRNA let-7. Lin28B overexpression in the PDAC cell lines had the opposite effect. In human PDAC samples, high Lin28B expression was associated with decreased let-7 expression and increased c-MYC, HMGA2 and KRAS expression. Thus, Lin28B is a novel marker for predicting the prognosis of patients with PDAC and might be a potential therapeutic target for PDAC.

## INTRODUCTION

Pancreatic ductal adenocarcinoma (PDAC) is one of the most lethal human malignancies, with an overall 5-year survival rate of less than 7% [1]. This dismal outcome is attributed to its late presentation and the early metastasis and dissemination of tumor cells [2]. Curative resection is the core of successful PDAC therapy, but only 15%-20% of patients are diagnosed during the early stages of the disease when surgical resection can be offered [3]. A large proportion of patients are diagnosed with locally advanced or metastatic disease at the time of presentation [1]. Adjuvant chemotherapy has been shown to improve the 5-year survival rate to 20%-25%, although the evidence remains controversial [4]. Therefore, PDAC remains a devastating disease, and the development of new diagnostic and treatment strategies for pancreatic cancer is urgently needed.

Lin28B is a homologue of Lin28 [5], a heterochronic gene that was initially described as a regulator of developmental timing in *Caenorhabditis elegans* [6, 7]. Lin28 and Lin28B each contain an N-terminal cold shock domain and a pair of retroviral-type CCHC zinc fingers near the C-terminus that confer RNA binding ability [7, 8] and inhibit the biogenesis of tumor-suppressive miRNAs of the let-7 family [9–11]. Although Lin28 and Lin28B share similar structures, they exhibit different functions [12, 13]. Lin28 is highly expressed in human embryonic stem cells and induces pluripotency when expressed in somatic fibroblasts in conjunction with three other factors (OCT4, SOX2, and KLF4) [14]. Lin28 is essential for the maintenance of the germline stem cell state and the regulation of miRNA activity during human ovary development [15]. Based on these findings, Lin28 is related to stem cell function. However, Lin28B, which was first identified in hepatocellular carcinoma [5], has important functions during the transformation of cells from an inflammatory to malignant state [16]. To date, Lin28 and Lin28B have been reported to be distinctively or exclusively expressed in several tumors, including hepatocellular carcinoma, esophageal cancer, oral squamous cell carcinoma, and colorectal cancer [17–20].

Lin28 and Lin28B are implicated in tumorigenesis in different cancers, but Lin28B is more frequently overexpressed in human cancers. Lin28B overexpression is observed in hepatocellular cancer (HCC) [21], breast cancer [21], lung cancer [21], colon cancer [22], esophageal cancer [18], melanoma [21], gastric cancer [23] and oral squamous cell carcinoma (OSCC) [19]. Increased Lin28B expression is correlated with a poor prognosis in patients with HCC, colon cancer, gastric cancer and esophageal cancer [21–23]. In addition, an analysis of Lin28 and Lin28B expression in various cancers suggested that Lin28B may be the more relevant homologue in tumorigenesis [21]. As shown in the recent study by Shao et al, Lin28B suppresses let-7b expression to promote human pancreatic cancer stem cell (PCSC) proliferation [24]. Nevertheless, the clinical significance of Lin28B and its correlation with the clinical features of the tumor and patients' clinical outcomes remain unclear. In this study, we explored the clinical implications and the underlying mechanisms of Lin28B in PDAC by examining tissues from patients with PDAC and performing experiments with PDAC cell lines. Lin28B expression was not only markedly upregulated in human PDAC tissues but was also well correlated with cancer metastasis and a poor prognosis. The usefulness of Lin28B as a prognostic factor was assessed in our study using a multivariate analysis. In addition, Lin28B overexpression promoted and Lin28B silencing abrogated cell proliferation, migration and the epithelial-mesenchymal transition (EMT). Lin28B decreased let-7 levels and activated several oncogenic pathways in PDAC cells. Thus, Lin28B is an important mediator of PDAC metastasis and an independent prognostic predictor in patients with PDAC.

## RESULTS

### Upregulation of Lin28B correlates with progression and a poor prognosis in patients with PDAC

**Figure 1:**
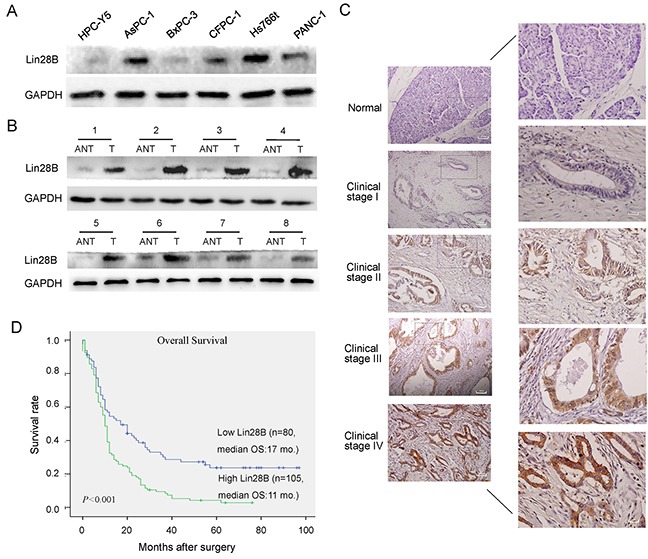
Lin28B expression is upregulated in PDAC cell lines and primary human PDAC tissues **(A, B)** Western blot analysis of Lin28B expression in an immortalized human pancreatic ductal epithelial cell line, 5 cultured pancreatic cancer cell lines, and 8 paired primary PDAC tissues (T) and matched adjacent non-tumor tissues (ANT) from the same patient. GAPDH was used as a loading control. **(C)** IHC staining for Lin28B in human PDAC tissue (clinical stage I–IV) compared with normal pancreatic tissue. **(D)** Kaplan-Meier curves of patients with PDAC and low or high Lin28B expression (n=182; P<0.001, log-rank test).

We first examined Lin28B expression in PDAC cell lines and human PDAC tissues to investigate the biological role of Lin28B in human PDAC progression. The Lin28B protein was markedly overexpressed in the PDAC cell lines (Figure 1A). Comparative analyses consistently showed that Lin28B expression was differentially elevated in 8 human PDAC samples and their matched adjacent non-tumor tissues (Figure 1B). The levels of the Lin28B protein were investigated in an additional 185 archived, formalin-fixed, paraffin-embedded PDAC tissues and 10 normal pancreatic tissues using immunohistochemistry (IHC). Lin28B was markedly upregulated in pancreatic cancer tissues but was nearly undetectable in normal pancreatic tissues (Figure 1C). Lin28B expression was strongly correlated with clinical stage, lymph node status, distant metastasis and vascular invasion in patients with PDAC (Table 1). According to the Kaplan-Meier survival analysis and log-rank tests, Lin28B overexpression was correlated with shorter overall survival (Figure 1D). The clinical stage and Lin28B expression were each recognized as independent prognostic factors for PDAC in the univariate and multivariate analyses (Table 2).

**Table 1:** Correlation between Lin28B expression and the clinicopathological characteristics of patients with pancreatic cancer

Characteristic	No. (n=185)	Lin28B expression		*P*^b^
		Low (n=80)	High (n=105)	
Gender				0.479
Male	121	53 (66.3%)	68 (64.8%)	
Female	64	27 (33.7%)	37 (35.2%)	
Age (years)				0.484
≤60	124	53 (66.3%)	71 (67.6%)	
>60	61	27 (33.7%)	34 (32.4%)	
Tumor location				0.320
Head	121	50 (62.5%)	71 (67.6%)	
Body/tail	64	25 (37.5%)	39 (32.4%)	
Tumor size (cm)				0.712
0-2	40	17 (21.2%)	23 (21.9%)	
2-5	116	50 (62.5%)	66 (62.9%)	
>5	29	13 (16.3%)	16 (15.2%)	
Tumor differentiation^a^				0.134
Well	17	9 (11.3%)	8 (7.6%)	
Moderate	114	54 (67.4%)	60 (57.2%)	
Poor	43	12 (15.0%)	31 (29.5%)	
Others	11	5 (6.3%)	6 (5.7%)	
Clinical stage				<0.001*
I-II	131	70 (87.5%)	61 (58.1%)	
III-IV	54	10 (12.5%)	41 (41.9%)	
Pathologic tumor status				0.003*
T1-T2	79	44 (55.0%)	35 (33.3%)	
T3-T4	106	36 (45.0%)	70 (66.7%)	
Lymph node status				0.026*
N0	121	59 (73.8%)	62 (59.0%)	
N1	64	21 (26.2%)	43 (41.0%)	
Distant metastasis				<0.001*
M0	160	77 (96.3%)	83 (79.0%)	
M1	25	3 (3.8%)	22 (21.0%)	
Perineural invasion				0.560
Absent	134	58 (72.5%)	76 (72.4%)	
Present	51	22 (27.5%)	29 (27.6%)	
Vascular invasion				0.038*
Absent	150	70 (87.5%)	80 (76.2%)	
Present	35	10 (12.5%)	25 (23.8%)	

^a^P value from χ^2^ or Fisher's exact test. *Statistically significant (P<0.05).

**Table 2:** Univariate and multivariate Cox regression analyses of different prognostic variables in patients with pancreatic cancer

Variable	Subset	HR (95%) CI^a^	*P*^b^
*Univariate analysis*			
Lin28B expression	High versus Low	1.915 (1.377-2.664)	<0.001*
Gender	Male versus Female	0.950 (0.807-1.118)	0.536
Age (years)	> 60 versus ≤60	1.271 (0.913-1.772)	0.155
Tumor location	Head versus Body/tail	1.912 (0.779-3.518)	0.253
Tumor size (cm)	>2 versus ≤2	1.772 (0.912-3.028)	0.475
Tumor differentiation	Poor/Moderate versus Well	1.487 (0.651-2.022)	0.511
Clinical stage	III + IV versus I + II	2.382 (1.678-3.383)	<0.001*
Pathologic tumor status	T3+T4 versus T1+T2	1.948 (1.404-2.703)	<0.001*
Lymph node status	N1 versus N0	1.625 (1.175-2.247)	0.003*
Distant metastasis	M1 versus M0	1.983 (1.276-3.081)	0.002*
Perineural invasion	Present versus Absent	1.691 (1.203-2.391)	0.003*
Vascular invasion	Present versus Absent	1.519 (1.032-2.236)	0.034*
*Multivariate analysis*			
Lin28B expression	High versus Low	1.475 (1.030-2.112)	0.034*
Clinical stage	III + IV versus I + II	1.683 (1.064-2.661)	0.026*
Pathologic tumor status	T3+T4 versus T1+T2	1.410 (0.961-2.069)	0.079
Lymph node status	N1 versus N0	1.218 (0.863-1.719)	0.261
Distant metastasis	M1 versus M0	0.982 (0.568-1.697)	0.947
Vascular invasion	Present versus Absent	0.954 (0.618-1.472)	0.831

HR, hazard ratio; CI, confidence interval.

^a^ 95% Wald confidence limits.

^b^ P value from Cox regression analyses. *Statistically significant (P < 0.05).

### Silencing of Lin28B inhibits pancreatic cancer cell proliferation and induces cell cycle arrest

**Figure 2:**
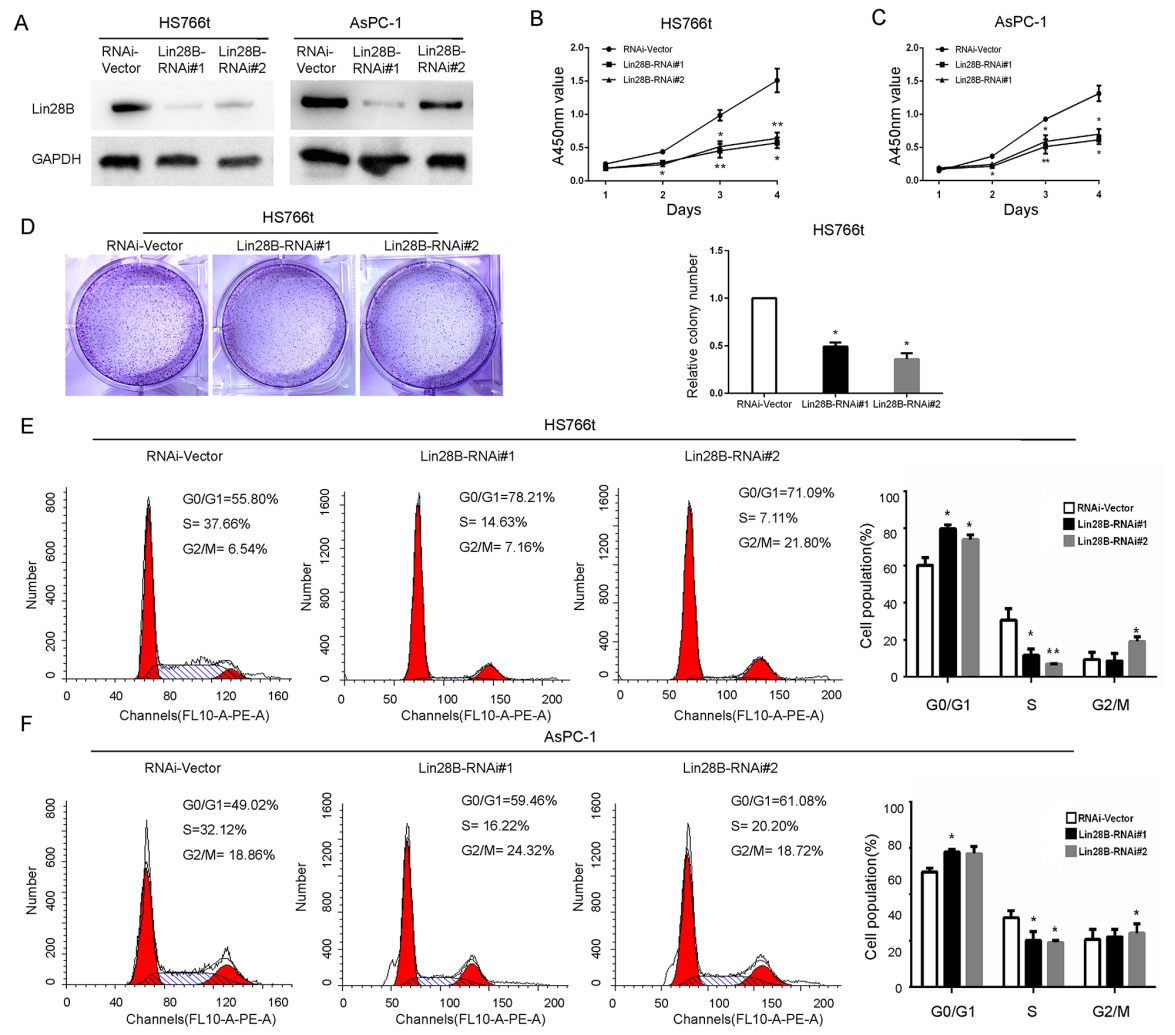
Lin28B silencing inhibits pancreatic cancer cell proliferation and induces cell cycle arrest **(A)** Western blot analysis of Lin28B expression in two PDAC cell lines stably transduced with lentiviral, short hairpin RNAs. GAPDH was used as a loading control. The results using the image j software to measure its gray value. **(B, C)** The effects of Lin28B silencing on pancreatic cancer cell growth were measured using a CCK-8 assay. The results are presented as the means±S.D. of the values obtained from three independent experiments. Statistical significance was calculated using Student's t-test. **(D)** Representative images and results of the Hs766t cell lines colony formation assay was summarized as means±S.D. of 3 independent experiments. *P <0.05. **(E, F)** Based on the flow cytometric analysis, Lin28B knockdown resulted in a significant increase in the percentage of AsPC-1 and Hs766t cells in G0/G1 phase, but a decrease in the percentage of cells in S phase. *P<0.05; **P<0.01.

Lin28B was silenced using a lentiviral infection system to establish two stable pancreatic cancer cell lines, AsPC-1 and Hs766t, both of which express high levels of Lin28B, to investigate whether Lin28B plays a role in the pathogenesis of PDAC (Figure 2A). According to the results of the cell counting kit-8 (CCK-8) assay, Lin28B knockdown with a specific siRNA markedly decreased the rates of AsPC-1 and Hs766t cell proliferation compared with that of the vector-transfected cells (Figure 2B and 2C). We found that Lin28B knockdown significantly decreased the numbers of Hs766t cells colony formation (Figure 2D). The functional impact of Lin28B on the cell cycle was analyzed using flow cytometry. Compared with the control, Lin28B knockdown significantly decreased the fraction of AsPC-1 and Hs766t cells in S phase and increased the fraction of cells in G0/G1 phase (Figure 2E and 2F).

### Lin28B knockdown inhibits pancreatic cancer cell migration

**Figure 3:**
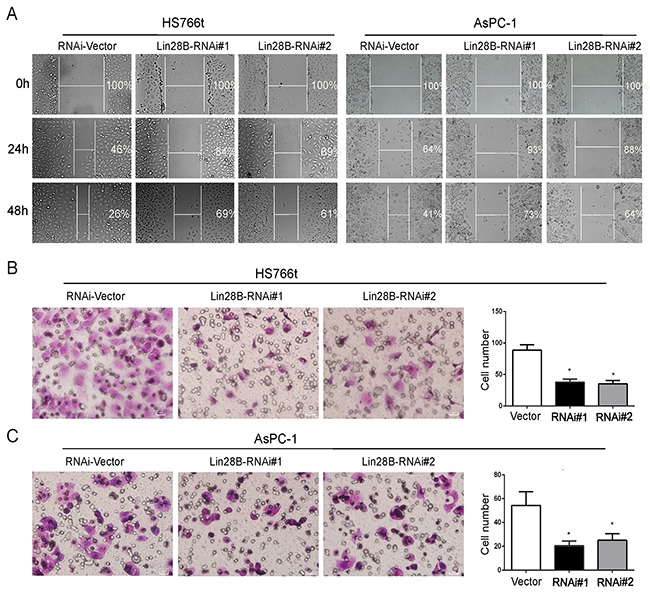
Lin28B silencing inhibits pancreatic cancer cell migration **(A)** Lin28B silencing disrupted wound closure in the wound-healing assay. The two cell lines were grown to confluence in six-well culture plates. Cell layers were scraped with a sterile pipet tip and incubated at 37°C. Photographs were taken at 24 and 48 h. **(B, C)** The inhibitory effect of Lin28B knockdown on AsPC-1 and Hs766t cell migration was confirmed using Transwell assays. Under the microscope, select 4 field of view to count and take the mean. The data are presented as the means±S.D. of three independent experiments. *P<0.05.

Since the Lin28B levels were significantly correlated with lymph node status and distant metastases in the PDAC samples, we examined the role of Lin28B in pancreatic cancer cell migration. Lin28B silencing noticeably decreased the migration of AsPC-1 and Hs766t cells in wound-healing assays in a time-dependent manner (Figure 3A). The Transwell migration assay yielded similar results, with a smaller number of cells penetrating the membrane in the Lin28B knockdown group than in the control group (Figure 3B and 3C).

### Lin28B overexpression promotes the aggressive phenotype of pancreatic cancer cells

**Figure 4:**
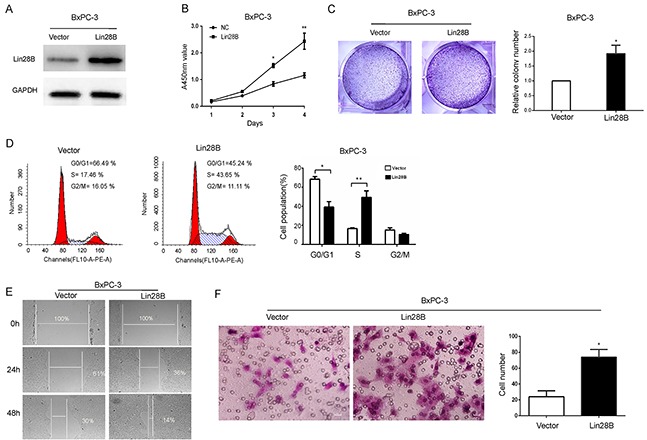
Lin28B promotes the aggressive phenotype of pancreatic cancer cells **(A)** Lin28B overexpression in the BxPC-3 cell line was analyzed by immunoblotting. GAPDH was used as a loading control. **(B)** Lin28B overexpression significantly promoted tumor proliferation, as detected by the CCK8 assay. **(C)** Representative image sand results of theBxPC-3 cell lines colony formation assay were summarized as means±S.D. of 3 independent experiments. *P <0.05. **(D)** Lin28B overexpression markedly accelerated the cell cycle transition, as determined by the flow cytometric analysis. **(E)** Lin28B promoted BxPC-3 cell migration. Images of wound-healing assays were captured after 0, 24 and 48 h. **(F)** The effect of Lin28B on BxPC-3 cell migration was confirmed using Transwell migration assays. Under the microscope, select 4 field of view to count and take the mean. The data are presented as the means±S.D. of three independent experiments. *P<0.05; **P<0.01.

Lin28B was ectopically expressed via a lentiviral infection system to establish stable BxPC-3 pancreatic cancer cells which normally express low levels of Lin28B, to determine the effect of Lin28B overexpression (Figure 4A). Consistent with the observations in Lin28B-silenced cells, ectopic expression of Lin28B significantly promoted cell proliferation, as measured by the CCK-8 assay (Figure 4B). Lin28B overexpression dramatically accelerated the transitions through the cell cycle and increased pancreatic cancer cell colony formation and migration (Figure 4C-4E).

### Downregulated Lin28B expression inhibits the EMT

**Figure 5:**
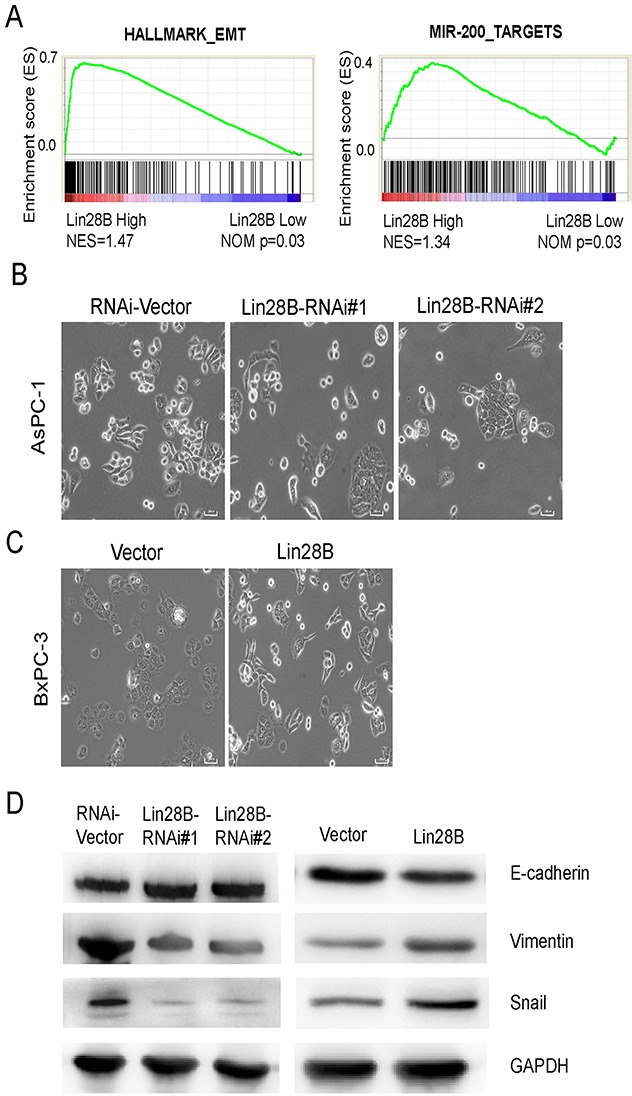
Lin28B silencing inhibits the EMT **(A)** GSEA plot of the expression of genes involved in the EMT pathway and miR-200 targets in the subgroups with high and low Lin28B expression Lin28B. **(B, C)** Inverse phase contrast microscopy of Lin28-silenced AsPC-1 cells and Lin28B-overexpressing BxPC-3 cells compared with the cells transfected with the corresponding vector control. **(D)** Western blot analysis of E-cadherin, Vimentin and Snail expression in Lin28-silenced AsPC-1 cells, Lin28B overexpressing BxPC-3 cells and cells transfected with the corresponding vector controls.

We further investigated the potential mechanisms by which Lin28B accelerates cancer metastasis. We used the Gene Expression Omnibus Series (GSE) data sets and divided pancreatic cancer specimens according to their relative Lin28B expression levels. Interestingly, subtypes with different Lin28B expression levels displayed distinct pathway profiles. The subtype with high Lin28B expression exhibited an average increase in the levels of genes involved in the EMT pathway and the expression of the targets of miR-200 (Figure 5A), an important EMT inhibitor [25, 26]. After 3 days of cell culture, the morphology of Lin28B-silenced AsPC-1 cells changed from single, spindle-shaped cells to a cobblestone-like epithelium. When the cells reached confluence, the Lin28B-silenced AsPC-1 cells exhibited more contact inhibition instead of the monolayer pattern observed in vector control cultures (Figure 5B). However, the opposite phenomenon occurred in Lin28B-overexpressing BxPC-3 PDAC cells (Figure 5C). These morphological changes were reminiscent of the EMT. In addition, the western blot analysis showed a reduction in the levels of the mesenchymal marker Vimentin and the transcription repressor Snail and increased expression of the epithelial marker E-cadherin in Lin28B-silenced AsPC-1 cells. We observed the opposite phenomena in Lin28B-overexpressing BxPC-3 cells (Figure 5D).

### Lin28B decreases let-7 levels and activates oncogenic pathways

**Figure 6:**
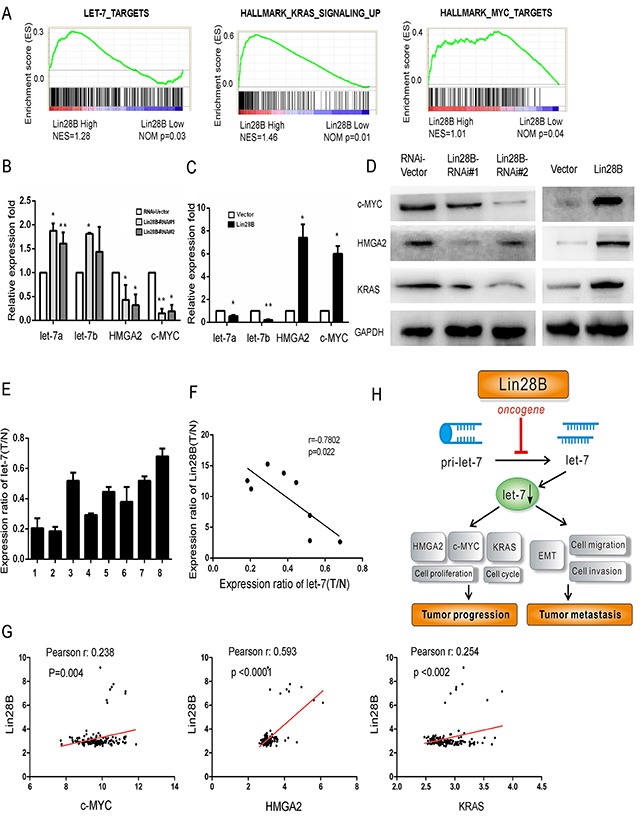
Lin28B overexpression decreases let-7 levels and activates oncogenic pathways GSEA plot of let-7, MYC targets and the KRAS signaling pathway in the subgroups with high and low Lin28B expression. **(B, C)** Results of the qRT-PCR analysis of the expression of the let-7a and let-7b miRNAs and c-MYC and HMGA2 mRNAs in Lin28B-silenced cells and Lin28B overexpressing BxPC-3 cells. **(D)** Western blot analysis showing the changes in the levels of the c-MYC, HMGA2 and KRAS proteins in the indicated cells. **(E)** The let-7a expression levels in the 8 pairs of tissues are indicated. **(F)** An inverse relationship between let-7a expression and the levels of the Lin28B protein was observed in PDAC tissues, as determined using Spearman's correlation analysis. **(G)** Analysis of Lin28B co-expression with MYC, HMGA2 and KRAS in pancreatic cancer tissues. The plotted data represent the log2 mRNA expression levels from the GSE data sets. **(H)** Illustrative model showing the proposed mechanism by which Lin28B facilitates the progression and metastasis of PDAC via downregulation of let-7 expression.

The most well-characterized function of Lin28B is to repress the biogenesis of a family of 12 tumor suppressor miRNAs, collectively referred to as let-7 [10]. We analyzed the GSE data sets to determine whether Lin28B promotes the growth and survival of PDAC cells by inhibiting let-7. The expression of let-7 targets was substantially increased in the subtype with high Lin28B expression, which exhibited increased levels of KRAS signaling intermediates and c-MYC targets (Figure 6A). Furthermore, the qRT-PCR analysis showed increased expression of let-7a and let-7b in Lin28B-silenced AsPC-1 cells (Figure 6B), whereas the opposite was observed in Lin28B-overexpressing BxPC-3 PDAC cells (Figure 6C). The levels of c-MYC, HMGA2 and KRAS were markedly increased in the western blots of Lin28B-overexpressing cells and decreased in the Lin28B-silenced cells compared with the levels in the control cells (Figure 6D). These observations prompted us to investigate the relevance of this pathway in clinical samples. The let-7a level was substantially reduced in the tumor tissues compared with that in the matched adjacent non-tumor tissues in the eight human PDAC samples (Figure 6E). Meanwhile, let-7a expression was inversely associated with the levels of the Lin28B protein (r=0.7802, *P*=0.022, Figure 6F). In addition, we analyzed the expression of the c-MYC, HMGA2 and KRAS mRNAs in the pancreatic cancer samples from the GSE data sets. Consistently, Lin28B expression levels were positively correlated with the expression levels of c-MYC, HMGA2 and KRAS (Figure 6G). Taken together, these results further support the notion that overexpression of Lin28B decreased let-7 levels and activated oncogenic pathways, thereby facilitating the progression and metastasis, and leading to poor prognosis in patients with PDAC (Figure 6H).

## DISCUSSION

Lin28B has recently been suggested to act as an oncogene, facilitating malignant transformation and tumor progression [16–18]. Aberrant regulation of the Lin28B and let-7 loop in human malignancies is reportedly involved in cancer development, contributing to cell transformation, metastasis, resistance to cell death, metabolic reprogramming, and tumor-associated inflammation [27, 28]. In the present study, Lin28B expression was not only markedly upregulated in human PDAC tissues but was also well correlated with cancer metastasis and a poor prognosis. In addition, Lin28B silencing in PDAC cells inhibited cell proliferation, cell cycle transition, migration and the EMT and increased the expression of the c-MYC, HMGA2 and KRAS genes, which are targeted by the cancer suppressor miRNA let-7. However, Lin28B overexpression had the opposite effect. Besides, Lin28B decreased let-7 levels and activated several oncogenic pathways in PDAC samples. These findings support the important roles of Lin28B in facilitating the progression and metastasis of PDAC.

The effects of Lin28 and Lin28B, which seem similar to the effects of an oncogene, are largely due to their abilities to inhibit the let-7 miRNA family [10]. Although Lin28 and Lin28B share similar structures, they function through distinct mechanisms to repress let-7 processing. Lin28 inhibits let-7 biogenesis by recruiting a non-canonical poly (A) polymerase (Zcchc11/TUT4) to suppress pre-let-7 maturation [29], whereas Lin28B blocks let-7 processing through a Zcchc11-independent mechanism. Lin28B functions by sequestering primary let-7 transcripts and repressing their processing by the Microprocessor [12]. This distinction derives from the differential subcellular localization of the two proteins; Lin28 is predominantly located in the cytoplasm, whereas Lin28B is specifically located in the nucleoli and contains functional nuclear localization signals. The results obtained in the present study were inconsistent with this localization pattern. Lin28B immunoreactivity was predominantly observed in the cytoplasm of PDAC cells, although nuclear staining was also observed in some cancer cells. Although Lin28B-mediated repression of let-7 expression does not depend on Zcchc11 in multiple cell types, Lin28B may locate in the cytoplasm and use Zcchc11/TUT4 to suppress let-7 biogenesis in certain context, including PDAC cells. The distribution of Lin28B is regulated by the cell cycle. Lin28B is predominantly located in the cytoplasm of G1 phase cells, whereas nuclear accumulation of Lin28B was observed in S phase and G2 phase cells [5]. Lin28B is localized in the cytoplasm of Huh7 hepatoma cells [5, 13]. The different distribution patterns of Lin28B in cancer cells and its mechanisms should be examined in more detail in the future.

Lin28B, acts as an oncogene and facilitates malignant transformation [27, 30]. Indeed, Lin28B expression has recently been shown to correlate with poor outcomes in patients with malignant diseases. For example, Lin28B is overexpressed in stage I and II colon cancers and correlates with reduced survival and an increased probability of tumor recurrence [22]. In ovarian cancer, the level of Lin28B expression is correlated with tumor stage and lymph node metastasis [31]. Sirtuin 6, a nicotinamide adenine dinucleotide (NAD)+-dependent histone deacetylase, is inactivated and accelerates PDAC progression and metastasis by upregulating Lin28B [32]. The results of this study revealed a strong correlation between Lin28B expression and the overall survival of patients with PDAC. According to the Kaplan-Meier analysis, patient survival is negatively correlated with the level of Lin28B expression, as patients with higher Lin28B expression had a shorter overall survival. Based on the results of the Cox regression analysis, Lin28B expression and the tumor lymph node metastasis (TNM) stage were independent prognostic predictors for patients with PDAC. The correlation between high Lin28B expression and a poor prognosis in patients with PDAC in this study is comparable with the results of the studies described above. Thus, Lin28B expression may be a clinically relevant prognostic marker for various malignancies, including PDAC.

Based on compelling evidence, miRNAs function as oncogenes and tumor suppressor genes that are involved in tumor development and progression through the regulation of target gene expression [33–36]. However, emerging data suggesting that miRNAs themselves are subjected to post-transcriptional regulation by other genes adds to this complexity. One of the best characterized examples is the negative feedback loop between the let-7 miRNA family and Lin28, in which Lin28 represses let-7 family biogenesis [37]. Lin28B is necessary and sufficient for MYC-mediated let-7 repression, and Lin28B has a key role in MYC-dependent cellular proliferation [38]. Lin28B overexpression correlates with reduced patient survival and promotes colon cancer metastasis and recurrence [22]. In this study, we revealed, for the first time, the direct roles of Lin28B in PDAC metastasis and the EMT, which is broadly believed to enhance invasion. Lin28B silencing in PDAC cells inhibited cell proliferation, migration and the EMT, whereas Lin28B overexpression exerted the opposite effect. Lin-28B overexpression increased the expression of the HMGA2, c-MYC and KRAS genes, which are targeted by the cancer suppressor miRNA let-7. High Lin28B expression was associated with decreased let-7 expression and increased HMGA2, c-MYC and KRAS expression in human PDAC samples. Of the known let-7 targets, HMGA2 is the most frequently reported target of let-7 involved in inhibiting invasion and metastasis [39]. HMGA2 was also reported to elicit the EMT by inducing the transcriptional activation of other EMT inducers, including Snail and Twist [40, 41]. Thus, Lin28B may increase the proliferation and migration of PDAC cells by directly inhibiting let-7 expression and subsequently upregulating HMGA2, c-MYC and KRAS expression.

Lin28/Lin28B and the let-7 family have recently been shown to exert opposite roles in many cellular processes, particularly in cancer development and progression [27]. Indeed, inverse expression of Lin28/Lin28B and let-7 is observed in normal and malignant tissues [18, 21]. In this study, Lin28B overexpression decreased let-7 levels and Lin28B expression was inversely correlated with let-7 expression in human PDAC samples. The presence of a double-negative feedback loop between Lin28/Lin28B and let-7 has also been reported [11, 28]. In the second feedback loop, Lin28/Lin28B depresses c-MYC by inhibiting let-7, and c-MYC transcriptionally activates Lin28/Lin28B [38, 42]. The third feedback loop involves Lin28B, let-7, NF-kB, and IL-6 [16]. NF-kB directly activates Lin28B transcription, leading to the inhibition of let-7 and expression of IL-6 (a let-7 target). In addition, IL-6 activates NF-kB, completing the positive feedback loop [16]. Therefore, Lin28 and let-7 may form a complex feedback loop during malignant transformation. However, Lin28 and Lin28B function through distinct mechanisms to suppress let-7 processing [12]. Further studies are needed to elucidate the roles of Lin28/Lin28B and the let-7 network in PDAC.

In conclusion, Lin28B is significantly overexpressed in human PDAC tissues and may serve as a novel prognostic biomarker. Lin28B participates in the EMT and represses the biogenesis of let-7, which may be one of the molecular mechanisms by which Lin28B promotes cancer progression and metastasis.

## MATERIALS AND METHODS

### Cell lines and treatments

The human pancreatic cancer cell lines AsPC-1, BxPC-3, CFPC-1, PANC-1 and Hs766t (ATCC, Manassas, VA, USA) and an immortalized human pancreatic ductal epithelial cell line (HPC-Y5, Chinese Academy of Sciences) were cultured in DMEM (Gibco, Grand Island, NY, USA) supplemented with 10% fetal bovine serum (Gibco) in a 37°C incubator with a humidified 5% CO_2_ atmosphere.

### Tissue specimens

One hundred eighty-five paraffin-embedded, pancreatic cancer samples and 10 normal pancreatic tissues were acquired from the archival collections of Southwest Hospital, Third Military Medical University, Chongqing, China. None of the patients had received radiotherapy or chemotherapy before surgery. The normal pancreatic samples were acquired from organ donors. In addition, eight fresh PDAC tissue samples and matched adjacent non-tumor tissue samples were collected from the same patient and frozen and stored in liquid nitrogen until further use. This study was approved and supervised by the ethical committee of Southwest Hospital.

### Western blot analysis

Western blotting was performed as previously described [43]. The following antibodies were used: Lin28B (Abcam, Cambridge, UK, ab71415), E-cadherin, Vimentin, Snail (Abcam, Cambridge, UK), GAPDH (Boster, Wuhan, China), c-MYC, HMGA2 and KRAS (Proteintech, Chicago, USA). Bands were visualized using an enhanced chemiluminescence (ECL) kit (Millipore, Billerica, MA, USA) according to the manufacturer's protocol. The results using the Image J software to measure its gray value.

### RNA isolation and quantitative real-time PCR (qRT-PCR)

Total RNA was extracted from tumor cells using RNAiso (TaKaRa, Dalian, China). Complementary DNA synthesis was performed using a PrimeScript RT reagent kit (TaKaRa), and qRT-PCR was performed with SYBR Premix Ex Taq II (TaKaRa) using a Stratagene Mx3000P real-time PCR system (Agilent Technologies, Santa Clara, CA, USA). The primer sequences are listed in Table 3.

**Table 3:** Sequences of the primer pairs used to analyze the genes of interest

Gene	Primer sequence
let-7a-F	5′- GCTGAGGTAGTAGGTTGTATAGTT-3′
let-7a-R	5′-GTGCAGGGTCCGAGGT-3′
let-7b-F	5′- GGGTGAGGTAGTAGGTTGTGTGGT-3′
let-7b-R	5′-GTGCAGGGTCCGAGGT-3′
U6-F	5′-TGGCACCCAGCACAATGAA-3′
U6-R	5′-CTAAGTCATAGTCCGCCTAGAAGCA-3′
HMGA2 -F	5′- ACCCAGGGGAAGACCCAAA -3′
HMGA2 -R	5′- CCTCTTGGCCGTTTTTCTCCA -3′
c-MYC -F	5′- GGCTCCTGGCAAAAGGTCA -3′
c-MYC -R	5′- CTGCGTAGTTGTGCTGATGT -3′
β-actin-F	5′-GACAGGATGCAGAAGGAGATTACT-3′
β-actin-R	5′-TGATCCACATCTGCTGGAAGGT-3′

### Immunohistochemistry and staining assessment

Immunohistochemistry was performed as previously described [43]. Primary antibodies against Lin28B were used. The staining intensity was scored as follows: 1, weak staining; 2, moderate staining; and 3, strong staining. The stained area was identified by determining the percentage of positively stained cells, which was scored as follows: 0, no staining; 1, 1-10% positively stained cells; 2, 10-50% positively stained cells; or 3, >50% positively stained cells. The immunohistochemical score (IS) for each slice was scored by multiplying the staining intensity and area scores to yield a final score ranging from 0 to 9. Samples with an IS≥6 were defined as expressing high Lin28B levels and samples with an IS<6 were defined as expressing low Lin28B levels.

### Lentivirus transductions

The Lin28B shRNA lentiviral particles consisted of the GV248 expression vector encoding the hU6-MCS-Ubiquitin-EGFP-IRES-puromycin precursor that produced both Lin28B-RNAi #1 and #2 (5-GCAGGCATAATAAGCAAGTTA-3#; 5#-GCCTTGAGTCAATACGGGTAA-3#). The lentiviral particles overexpressing Lin28B contained the GV358 expression vector encoding the Ubi-MCS-3FLAG-SV40-EGFP-IPES-puromycin precursor and Lin28B (GENE_ID:389421; GenBank: NM_001004317) purchased from GeneChem (Shanghai, China). The lentiviruses were transduced into PDAC cells according to the manufacturer's instructions. The GFP-positive virus-infected cells were selected using puromycin.

### Colony formation assays

For the colony formation assay, 1 × 10^3^ cells were seeded and cultured in six-well plates for 10 days until visible colonies were formed. Survival colonies were fixed and stained with Fast Richie dye (Jiancheng Biotech, Nanjing, China). Colonies containing more than 100 cells were counted. The experiments were repeated three times.

### Cell proliferation and cell cycle analyses

Cell proliferation efficiency was measured using (2-(2-methoxy-4-nitrophenyl)-3-(4-nitrophenyl)-5-(2,4 disulfophenyl)-2H-tetrazolium, monosodium salt) (WST-8) staining with a CCK-8 assay (Dojindo, Shanghai, China) according to the manufacturer's instructions. Cells (2,000/well) were seeded into 96-well plates. The cells were collected and fixed overnight at −20°C in 70% ethanol and then stained with propidium iodide (Kaiji, Nanjing, China) in a phosphate-buffered saline solution containing RNase, according to the manufacturer's instructions. All data were analyzed using ModFit software (BD Biosciences, MD, USA).

### Wound-healing assay and migration assay

The wound-healing and migration assays were performed using previously described methods [44]. Cell migration was measured using a Transwell chamber (24-well insert, 8-μm pore size, Millipore, Massachusetts, USA). After passing through the gap. Under the microscope, selected 4 field of view to count and taken the mean.

### Gene set enrichment analysis (GSEA)

A GSEA was performed to obtain further insights into the Lin28B-mediated biological pathways involved in the pathogenesis of pancreatic cancer. Human whole genome microarray datasets GSE15471/16515/32676/32688/42952/9599/71989 for pancreatic cancer and normal adjacent tissues were downloaded from the Gene Expression Omnibus (GEO). All datasets were analyzed using Affymetrix U133 plus 2.0 chips and included 146 pancreatic cancer specimens. Raw data processing, quality control and normalization were performed using previously described methods [45, 46]. GSEA was performed using the java GSEA Desktop Application (Broad Institute) with the hallmark gene sets (n=50) and miRNA targets sets (n=221) implemented in the Molecular Signatures Database (MsigDB, http://software.broadinstitute.org/gsea/msigdb). Expression and phenotype data were formatted according to the user guide.

### Statistical analyses

All data were analyzed using SPSS 18.0 statistical software (version 18.0, Chicago, IL, USA). The data are presented as the means±S.D. deviations (S.D.) of three independent experiments. Correlations between Lin28B expression and clinicopathological parameters were evaluated using χ^2^ and Fisher's exact tests. The statistical tests were two-sided, and *P* values<0.05 were considered significant. Survival curves were generated according to the Kaplan-Meier method, and statistical analysis was performed using the log-rank test. The Cox proportional hazards regression model was used to identify independent prognostic factors. Calculations were performed using GraphPad Prism Software (www.graphpad.com).
